# Investigation of material removal in inner-jet electrochemical grinding of GH4169 alloy

**DOI:** 10.1038/s41598-017-03770-1

**Published:** 2017-06-14

**Authors:** Hansong Li, Shen Niu, Qingliang Zhang, Shuxing Fu, Ningsong Qu

**Affiliations:** 1 College of Mechanical and Electrical Engineering, Nanjing University of Aeronautics and Astronautics, Nanjing, 210016 China; 2 AVIC Jincheng Nanjing Engineering Institute of Aircraft Systems, Nanjing, 211106 China

## Abstract

Electrochemical grinding (ECG) is a low-cost and highly efficient process for application to difficult-to-machine materials. In this process, the electrolyte supply mode directly affects machining stability and efficiency. This paper proposes a flow channel structure for an abrasive tool to be used for inner-jet ECG of GH4169 alloy. The tool is based on a dead-end tube with electrolyte outlet holes located in the sidewall. The diameter and number of outlet holes are determined through numerical simulation with the aim of achieving uniform electrolyte flow in the inter-electrode gap. Experiments show that the maximum feed rate and material removal rate are both improved by increasing the diamond grain size, applied voltage, electrolyte temperature and pressure. For a machining depth of 3 mm in a single pass, a feed rate of 2.4 mm min^−1^ is achieved experimentally. At this feed rate and machining depth, a sample is produced along a feed path under computer numerical control, with the feed direction changing four times. Inner-jet ECG with the proposed abrasive tool shows good efficiency and flexibility for processing hard-to-cut metals with a large removal depth.

## Introduction

GH4169 (Ni–Fe–Cr) alloy, with its excellent fatigue resistance, high-temperature strength, and good resistance to corrosion and radiation damage, is widely used in a number of important industries, including aerospace, navigation and petroleum^1–4^. However, its high strength and stiffness, low thermal conductivity, and tendency to high work hardening with consequent damage to tool materials make it extremely difficult to cut^5, 6^. When traditional cutting processes are used, it is impossible to avoid problems such as severe tool wear, high work hardening and low material-removal rate (MRR), all of which lead to unacceptably high manufacturing costs^7^. Hence, as with other difficult-to-cut materials, attention has increasingly been focused on the use of non-conventional processes to machine GH4169 alloy at low cost and with high efficiency.

Various non-traditional machining techniques based on a number of different principles have been developed to process difficult-to-cut materials, including electrical-discharge machining (EDM), laser-beam machining (LBM) and electrochemical machining (ECM), among others. Yadav and Yadava^8^ optimized the selected input process parameters for MRR, average surface roughness and average circularity of holes made by EDM in a nickel-based superalloy. Darwish *et al*.^9^ used LBM in both dry and wet media to fabricate channels in Inconel 718 and investigated the effects of laser power, pulse repetition rate and laser scan speed on channel width, depth and taper. Mohanty *et al*.^10^ used an analysis-of-variance approach to study the influence of various process parameters on performance characteristics such as MRR and surface roughness during ECM of Inconel 825.

Because of the mechanism by which it removes material, ECM has a significant advantage over both EDM and LBM in that it can produce machined workpieces without heat-affected zones and recast layers^11, 12^. However, ECM with a passive aqueous electrolyte suffers from the major disadvantage that a non-conductive passivation layer is formed on the machined surface of the metal, which can prevent further electrolytic dissolution of the anodic workpiece^13–15^. Electrochemical grinding (ECG) is a non-conventional hybrid process based on a combination of ECM and mechanical grinding (MG)^16^, with the electrochemical and abrasive actions contributing about 90% and 10%, respectively, of the total material removal^17, 18^. In ECG, the abrasive action of a grinding wheel continually removes the soft passivation layer, thus exposing fresh metal for electrolytic reaction^14, 15, 19^. Furthermore, in contrast to MG alone, there is low tool wear and an absence of a hardened surface layer or surface distortions^20, 21^. When machining tough alloys, the MRR with ECG may be 5–10 times that achievable with conventional machining^22^. Hence, ECG is a technologically and economically viable approach for the fabrication of any electrically conductive difficult-to-cut material.

ECG uses a grinding tool made of metal to which abrasive is bonded, with an additional nozzle arrangement. Typically, a small removal depth and a high feed rate are adopted, with the aim of improving the surface integrity of difficult-to-cut materials. For example, Goswami *et al*.^21^ explored ECG of an alumina–aluminium interpenetrating-phase composite and found that the minimum surface roughness was obtained with a cutting depth of 0.08 mm and a feed rate of 24 mm min^−1^. Hascalık and Caydas^23^ used ECG with a cutting depth of 0.05 mm and a feed rate of 24 mm min^−1^ to remove the damaged surface layers of Ti6Al4V alloy machined by EDM. To enhance processing flexibility, computer numerical control (CNC) has been applied to ECG. Curtis *et al*.^24^ investigated a method for machining both sides of a V-shaped slot in a nickel-based superalloy by using a mounted grinding point with fir-tree geometry, with a depth of cut and feed rate of 0.5 mm and 10 mm min^−1^, respectively. Qu *et al*.^25^ used a spherical abrasive tool with rod-like geometry to machine Inconel 718, with a feed rate of 6.6 mm min^−1^ and a machining depth of 0.5 mm.

However, when the electrolyte is provided through an additional nozzle, most of it drains away from the workpiece surface instead of flowing into the narrow machining gap, which causes difficulties when it is desired to remove a large depth of material. To overcome this problem when machining GH4169 alloy, Zhang *et al*.^26^ proposed a method of inner-jet ECG using an abrasive tool based on a tube with a round end from which the electrolyte was ejected through seven outlet holes. In this pioneering work, a machining depth of 3 mm was achieved in just a single pass at a feed rate of 1.8 mm min^−1^. If the product of the removal depth and the feed rate is taken as a reflection of the machining area per unit time, it is clear that, compared with ECG using an additional nozzle, this inner-jet ECG method allows more efficient removal of greater depths of material. Even so, the positioning of the electrolyte outlet holes in the round end of the tool still imposes a limitation on the achievable feed rate: because the tool sidewall plays a major role in material removal at large depths, greater feed rates should be possible if the outlet holes are located in the sidewall of the tool. To determine the optimum number, positions and diameter of these holes, it is necessary to analyse the flow-channel structure of the ECG system.

Thus, to further improve the MRR and widen the applicability of inner-jet ECG, this paper proposes a flow-channel structure for an abrasive tool based on a dead-end tube, with electrolyte outlet holes located in the tool sidewall. The diameter and number of these holes are optimized by means of a finite-element fluid simulation method. Furthermore, the possibility of achieving a higher feed rate and MRR is explored experimentally on GH4169 alloy. Finally, a sample is produced under CNC using the proposed abrasive tool with optimized structure and a higher feed rate.

## Flow-field analysis

### Principle of the inner-jet ECG

**Figure 1:**
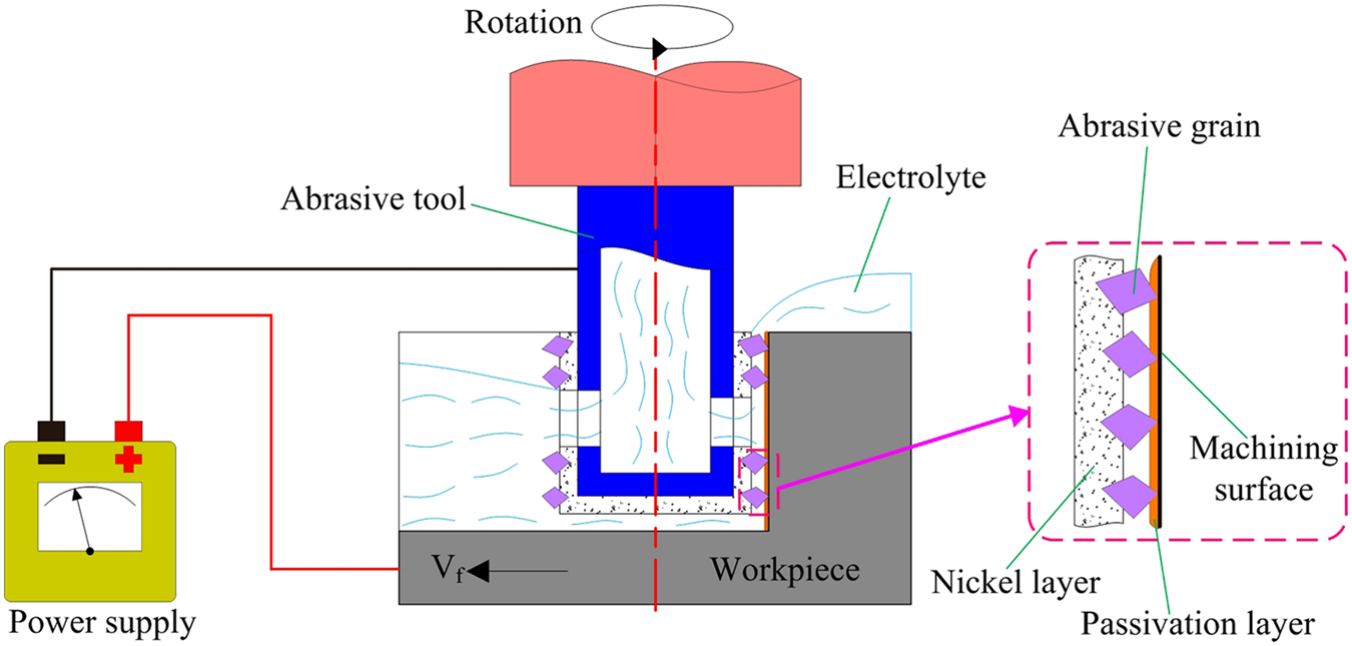
Schematic diagram of inner-jet ECG with the proposed abrasive tool.

Figure 1 is a schematic depiction of the principle of inner-jet ECG with the proposed abrasive tool. The latter is a tubular electrode with a flat dead-end, coated with electroplated diamond particles on its outer surface and with electrolyte outlet holes in the sidewall. In the machining process, the abrasive tool is charged negatively and the workpiece is connected to the positive pole of the power supply. Electrolyte is sprayed under pressure onto the machining surface of the workpiece through the outlet holes. The machining by-products and Joule heat are rapidly removed from the inter-electrode gap by the flow of electrolyte. As a result, the risk of blockage in the machining gap is significantly reduced and the process can start from the sidewall of the workpiece, thereby allowing the required depth to be achieved one pass. In addition, when the rotating abrasive tool is in contact with the moving workpiece, the protruding diamond particles continuously remove the passivation layer on the machining surface. With the use of CNC technology, more complicated structures can be produced by controlling the feed path of the workpiece.

### Physical models of flow field

The machining depth of the abrasive tool is 3 mm in a single pass. The outer diameter, inner diameter and wall thickness of the tool tube are 6, 4 and 1 mm, respectively. To obtain a uniform concentration of the machining by-products in the inter-electrode gap, the electrolyte outlet holes are uniformly distributed along the circumference of the tube. The axial distance from the bottom of the tube to the centreline of the outlet holes is 1.5 mm.

**Figure 2:**
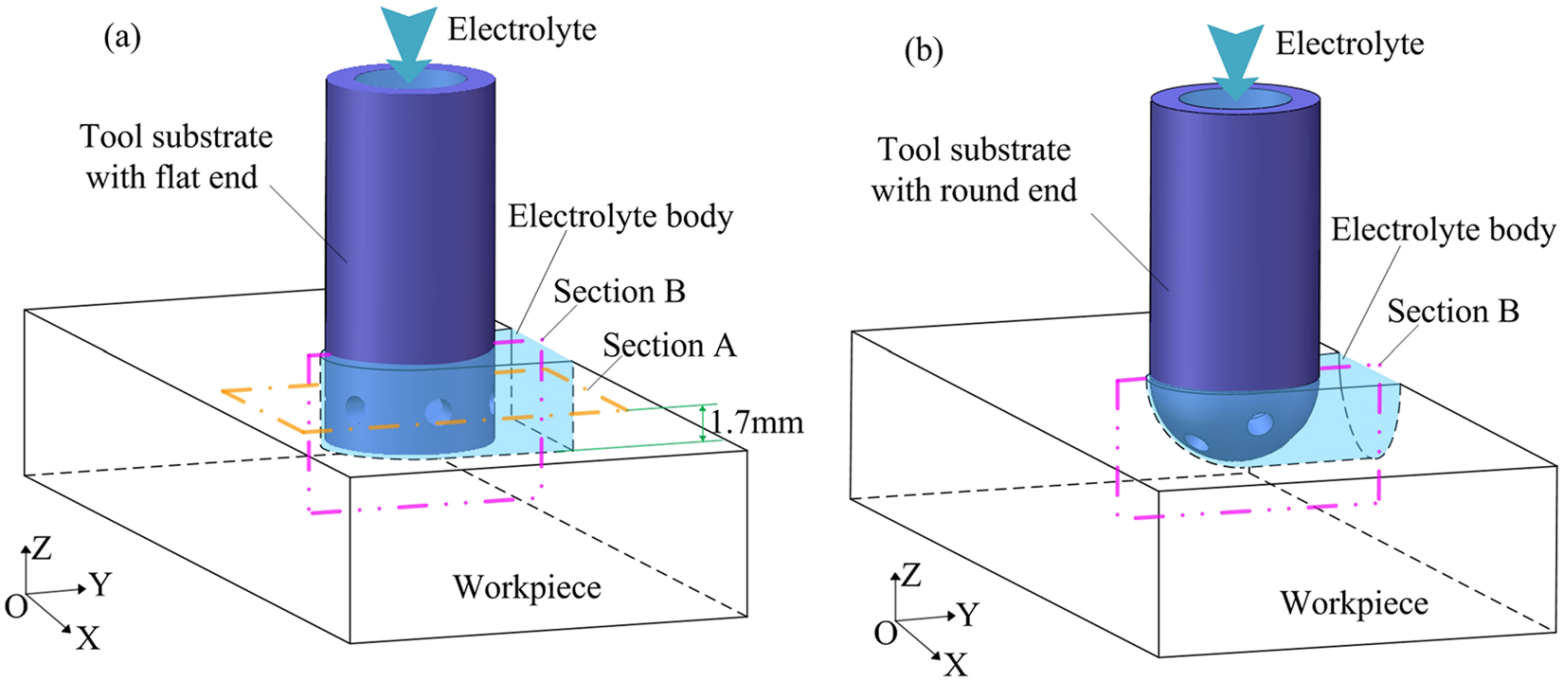
Electrolyte bodies in the flow-field simulation: (**a**) flat-ended tool tube; (**b**) round-ended tool tube.

To optimize the diameter and number of the outlet holes, a flow-field model is established for the flat-ended tube, as shown in Fig. 2a. Two values are considered for the hole diameter (0.6 and 1 mm) and two values for the number of holes (4 and 6). Section A is chosen in order to observe the different flow velocity distributions. In addition, another flow-field model is established for a tool based on a tube with a round end, as shown in Fig. 2b. Such an abrasive tool with a round end with seven outlet holes of diameter 1 mm was used in a previous study, in which a machining depth of 3 mm was achieved in one pass^26^. Section B is chosen in the same position in both flow-field models in order to compare the flow-velocity distributions in the inter-electrode gap.

To evaluate the flow-field distribution in each model, the following assumptions are made:

(i) The electrolyte flow is incompressible and continuous.
(ii) Energy dissipation and changes in electrolyte temperature are ignored.
(iii) The ECM process is taken to be an equilibrium state.
(iv) The flow motion is constrained by mass and momentum conservation.

On the basis of these assumptions, a computational fluid dynamics (CFD) model can be established. For finite-element analysis, the mass conservation and momentum conservation equations are

1 $$\rho \nabla \cdot u=0,$$

2 $$\rho (u\cdot \nabla )u=\nabla \cdot \{-pI+(\mu +{\mu }_{T})[\nabla u+{(\nabla u)}^{{\rm{T}}}]-\tfrac{2}{3}\rho KI\}+\rho g,$$

where *p* is the pressure, *ρ* is the density of the electrolyte, *μ* is its dynamic viscosity, *u* is the velocity vector along the *x*-axis, and *g* is the acceleration due to gravity.

Many experimental results have shown that turbulent flow is necessary for the stability of the ECM process^27^. Here, the three-dimensional electrolyte flow field is described using the standard *κ–ε* turbulence model^28^. The equations for the turbulence kinetic energy *κ* and dissipation rate *ε* are

3 $$\rho (u\cdot \nabla )k=\nabla \cdot [(\mu +\frac{{\mu }_{T}}{{\sigma }_{K}})\nabla k]+{\rho }_{K}-\rho \varepsilon ,$$

4 $$\frac{\partial }{\partial t}(\rho \varepsilon )+\frac{\partial }{\partial {x}_{i}}(\rho \varepsilon {u}_{i})=\nabla \cdot [(\mu +\frac{{\mu }_{T}}{{\sigma }_{\varepsilon }})\nabla \varepsilon ]+{C}_{C1}\frac{\varepsilon }{K}{\rho }_{K}-{C}_{C2}\rho \frac{{\varepsilon }^{2}}{K},\,\varepsilon =ep,$$

5 $${\mu }_{t}=\frac{\rho {C}_{\mu }{K}^{2}}{\varepsilon },\,{\rho }_{K}={\mu }_{T}\nabla u:[\nabla u+{(\nabla u)}^{{\rm{T}}}],$$

where *ρ*_*K*_ is the generation of turbulent kinetic energy *κ* due to mean velocity gradients, *μ*_*t*_ is the turbulent viscosity, and *σ*_*K*_ *, σ*_*ε*_ *, C*_*C*1_, *C*_*C*2_, *C*_*μ*_ and *K* are constants in the model (*σ*_*K*_ = 1.0, *σ*_*ε*_ = 1.3, *C*_*C*1_ = 1.44, *C*_*C*2_ = 1.92, *C*_*μ*_ = 0.09 and *K* = 0.41).

The boundary conditions for the numerical simulation are determined by setting values for the inlet and outlet pressures, which are taken to be 0.5 and 0.1 MPa, respectively. Based on the above equations and conditions, the flow-field simulation for each electrolyte body for the two flow-field models is conducted using the CFD software ANSYS Fluent 15.

### Results of numerical simulation

**Figure 3:**
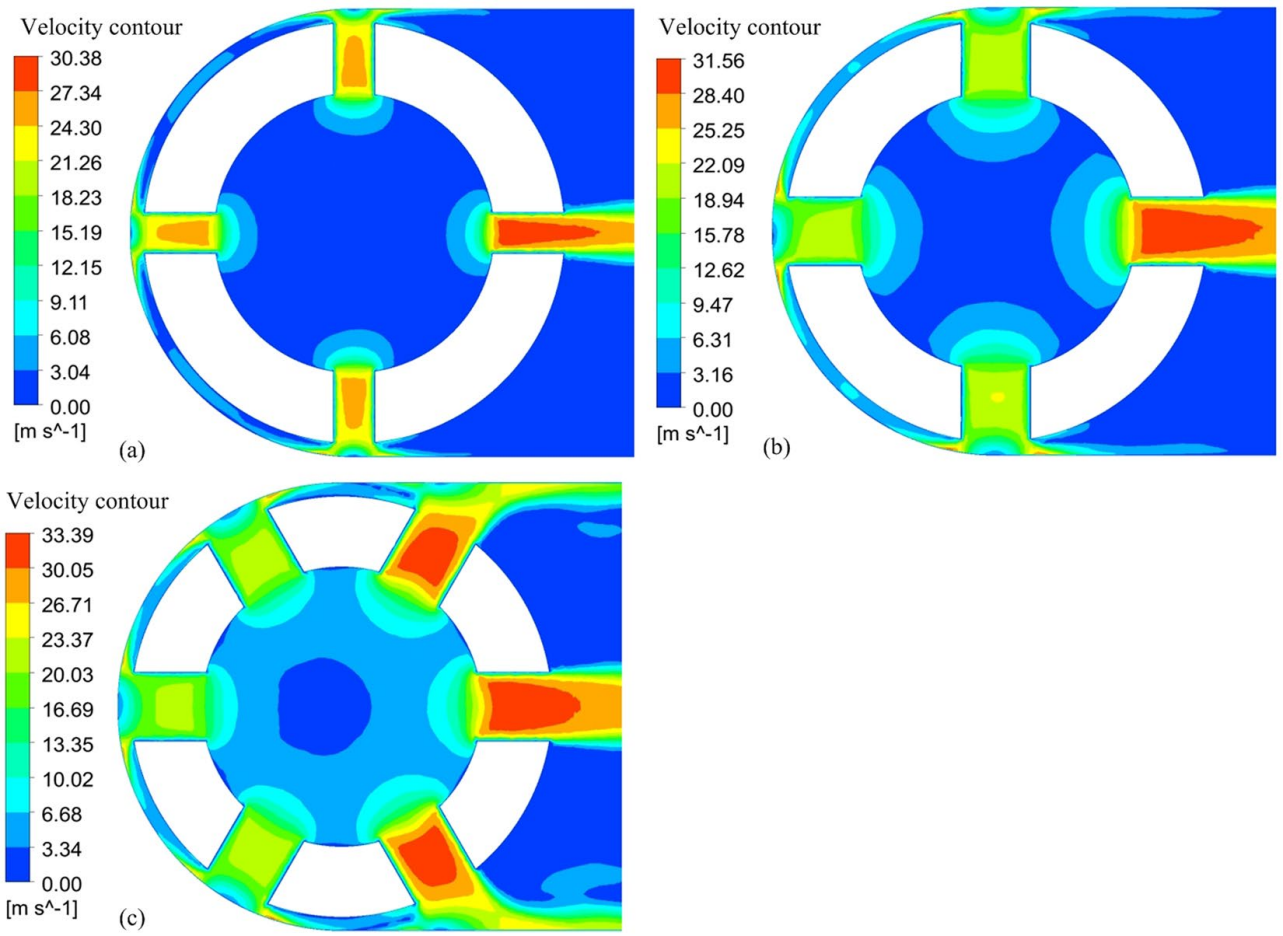
Velocity distributions at section A for a flat-ended tool tube: (**a**) four outlet holes of diameter 0.6 mm; (**b**) four outlet holes of diameter 1 mm; (**c**) six outlet holes of diameter 1 mm.

The simulation results for the flat-ended tool tube are shown in Fig. 3. A very large difference in flow velocity is observed between the outlet holes on the left and right sides because of the high fluid resistance in the inter-electrode gap, which leads to a non-uniform flow field and unstable machining in the inter-electrode gap. When a tool tube with four outlet holes of diameter 0.6 mm is used, there is an obvious dead-water zone in the inter-electrode gap (Fig. 3a). When the diameter of the outlet holes is increased to 1 mm, although the average flow velocity increases in the inter-electrode gap, there is still an obvious low-velocity zone between the two outlets (Fig. 3b). With an increase in the number of outlets to six, there remain only a few low-velocity zones in the inter-electrode gap (Fig. 3c). In an actual machining process, because the tool continues to rotate, the low-velocity zones are quickly refreshed. The simulation results indicate that the uniformity of the flow field in the inter-electrode gap can be greatly improved by increasing the diameter and number of outlet holes. However, this also decreases the effective working area of the tool, so further increases in the diameter and number of outlets will not be considered here. Therefore, a flat-ended tool with six outlet holes of diameter 1 mm is chosen as the optimal structure.

**Figure 4:**
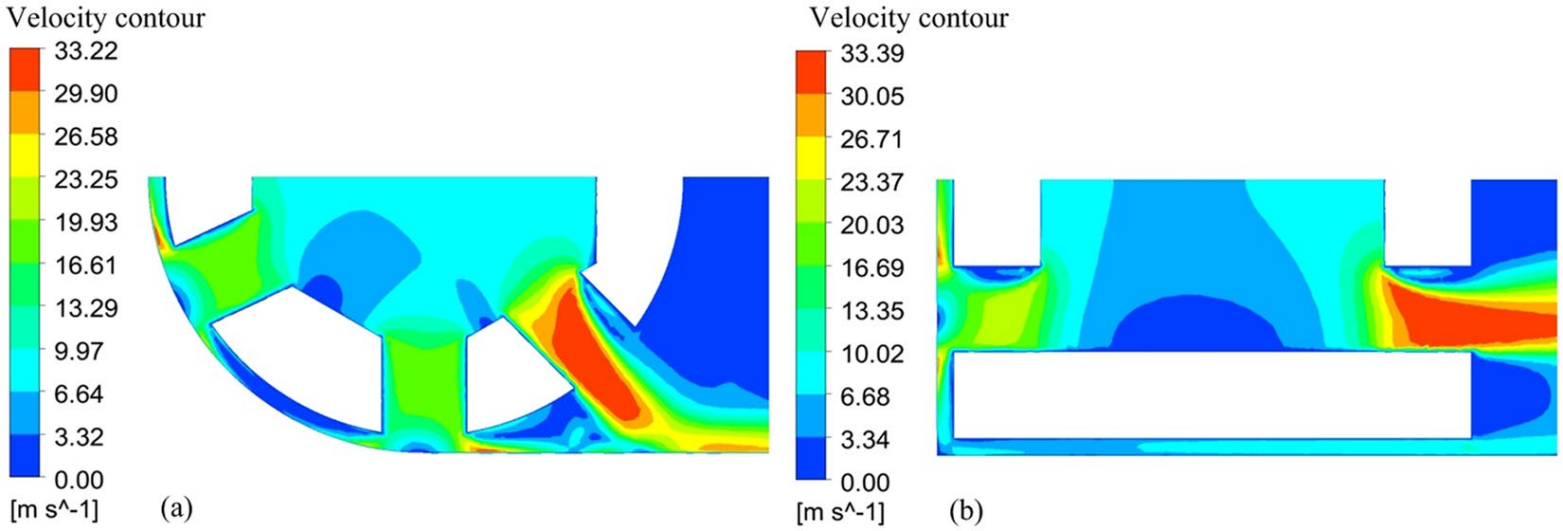
Velocity distributions at section B: (**a**) round-ended tool tube; (**b**) flat-ended tool tube.

Figure 4 presents velocity contours in section B using the two different tool tubes. For the round-ended tube, there is an obvious dead-water region in the front-end gap, which gives rise to a very uneven flow rate distribution (Fig. 4a). This can cause a non-uniform material dissolution rate and a non-uniform concentration of machining by-products. Hence, it is difficult to achieve a high feed rate with the round-ended tool tube. However, for the flat-ended tool tube with optimized outlet-hole number and diameter, some of the electrolyte flows from the front-end gap to the bottom gap, which leads to a more uniform flow velocity in the front-end gap (Fig. 4b). These contrasting results indicate that the optimized flat-ended tool tube provides better flow-channel properties than the round-ended tool tube.

### Experimental procedure

**Figure 5:**
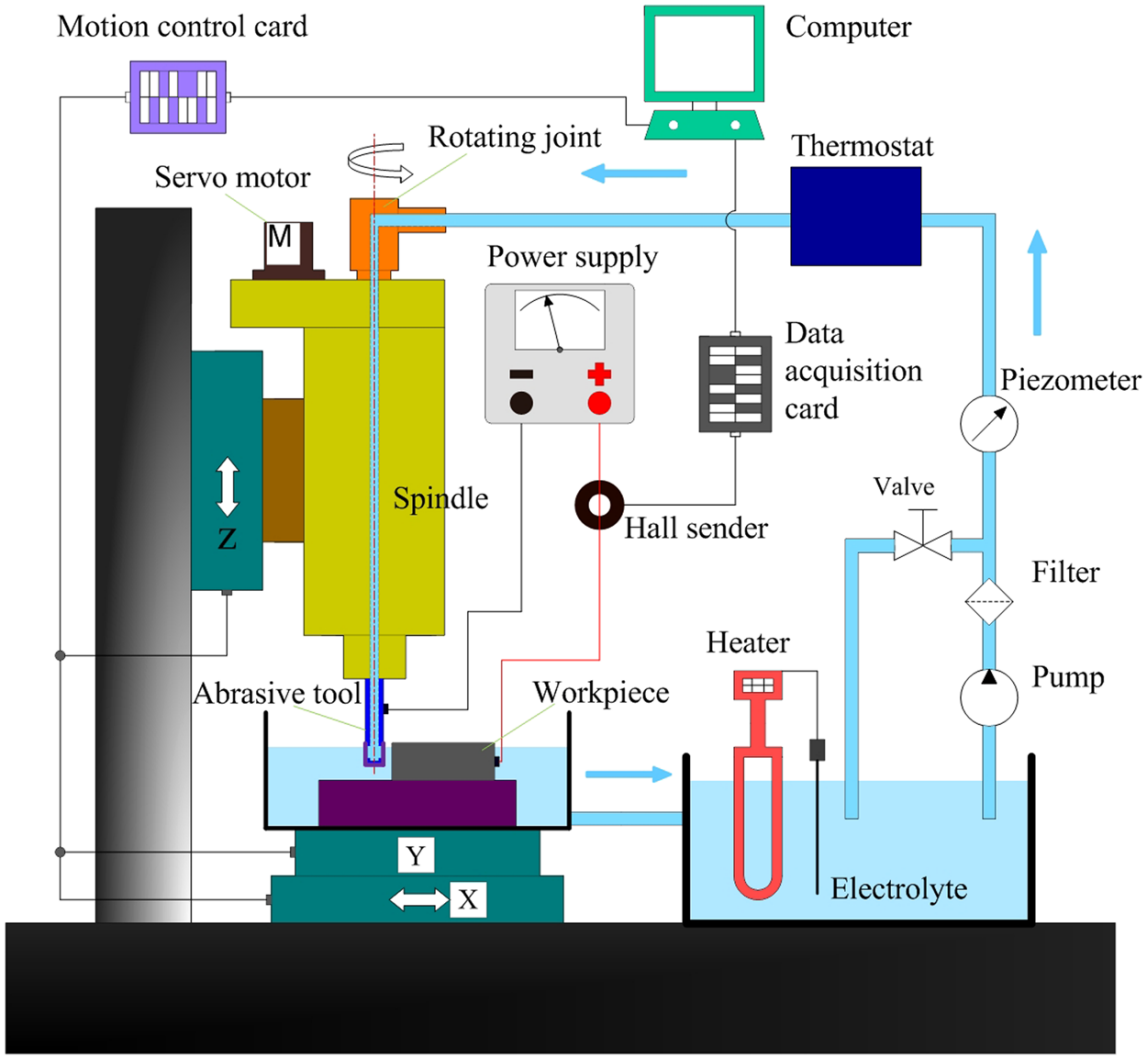
Schematic diagram of the machining setup.

Figure 5 shows a schematic view of the machining system. A rotating joint is connected to the top of the hollow spindle, and a bearing and dynamic seal are located between the inner and outer rings of this joint. The inner ring can rotate with the rotating spindle while the outer ring remains at rest. The filtered electrolyte, supplied from a pump, is injected into the working gap directly through the rotating joint, hollow spindle and inner holes of the abrasive tool. The abrasive tool is clamped to the end of the spindle and rotates with it. The workpiece is fed along a programmed trajectory via a motion control system, through which the feed rate can be changed and measured. A current detection unit is used to monitor the machining current, which is considered to be indicative of machining stability. The working voltage is applied between the cathode and anode through a DC power supply. A heater is used in the electrolyte tank to control the electrolyte temperature, which is maintained at the required level by means of a thermostat. The electrolyte pressure is adjusted by a valve and is measured by a piezometer.

The MRR is one of the most important criteria determining machining efficiency and is defined as

6 $${\rm{MRR}}=\frac{m}{t},$$

where *m* is the mass removed by ECG and *t* is the total processing time.

According to the theory of ECM, the inter-electrode gap Δ can be expressed as^27, 29^

7 $${\rm{\Delta }}=\frac{\eta \omega {\kappa }_{0}(U-\delta E)}{{v}_{f}},$$

where *η* is the current efficiency, *ω* is the volume electrochemical equivalent, *κ*_0_ is the electrolytic conductivity, *U* is the applied voltage, *δE* is the polarization potential and *v*_*f*_ is the feed rate.

From equation (7), it can be seen that the applied voltage and those electrolyte parameters that affect the conductivity, such as pressure and temperature, are the key factors determining the electrochemical action. The diamond grain size is also a critical factor, because it can influence the grinding action. Therefore, the experimental investigation will be conducted in terms of these parameters.

The feed rate is a major concern in industrial production^30^. According to equation (6), a greater MRR can be achieved by increasing the feed rate, because of the resulting shorter processing time. However, according to equation (7), an excessive feed rate would lead to too small a machining gap, which would impede refreshment of electrolyte and cause a short circuit or undesired machining^30^. Hence, it is relevant to attempt to determine the maximum feed rate in inner-jet ECG using the proposed abrasive tool.

**Figure 6:**
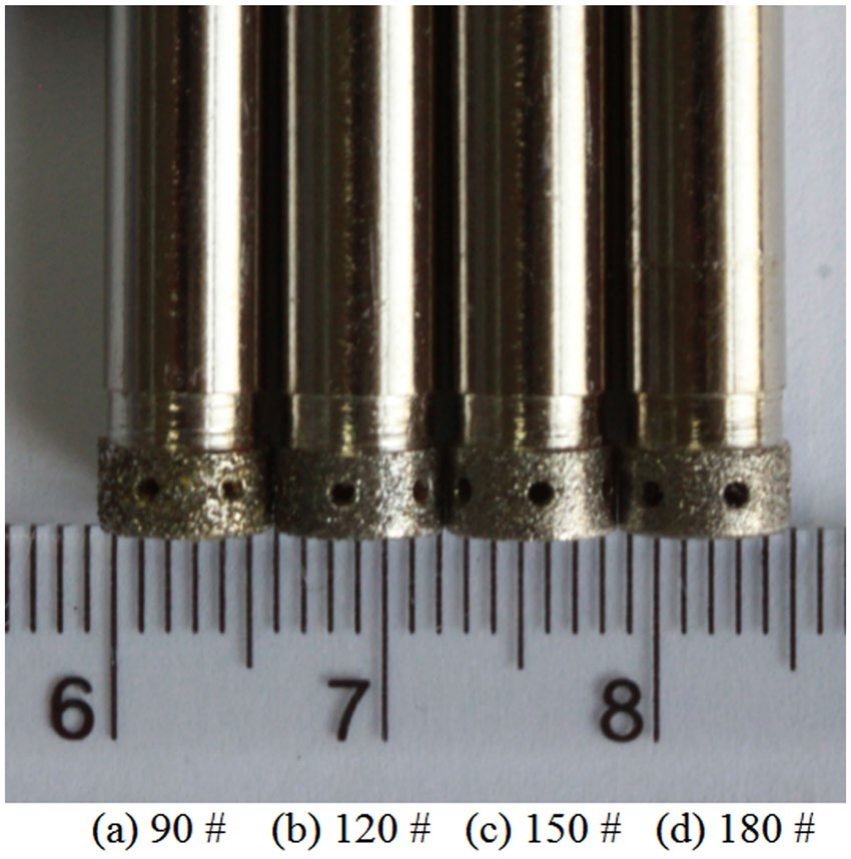
Four abrasive tools with different diamond grain sizes.

GH4169 workpieces with dimensions of 80 mm × 60 mm × 10 mm were weighed before and after each experiment by an analytical balance with a precision of 0.001 g. Cross-sectional images of machined slots were obtained using a three-dimensional profilometer (DVM5000, Leica, Germany). The machining conditions were as shown in Table 1. During the manufacturing process, the abrasive tool rotated continuously with the spindle at 1000 rev min^−1^. The electrolyte was an aqueous solution of sodium nitrate with a mass fraction of 10%. The diamond grain size of the tool ranged from 90 to 180# in four equally spaced intervals (Fig. 6). The applied voltage ranged from 10 to 25 V in four equally spaced intervals. The electrolyte temperature was adjusted from 20 to 35 °C in four equally spaced intervals. The electrolyte pressure was set from 0.2 to 0.5 MPa in four equally spaced intervals.

**Table 1:** Machining conditions^25, 26^.

Electrolyte	NaNO_3_ aqueous solution
Concentration	10 wt%
Spindle speed	1000 rev min^−1^
Diamond grain size	90, 120, 150, 180#
Applied voltage	10, 15, 20, 25 V
Electrolyte temperature	20, 35, 30, 35 °C
Electrolyte pressure	0.2, 0.3, 0.4, 0.5 MPa
Machined depth	3 mm

### Experimental results and discussion

The maximum feed rate was first tested for each group of machining parameters. Each test started with a low feed rate, which was then gradually increased by the motion control program, until a short-circuit current appeared in the current detection unit. Each test was repeated three times in order to confirm the same maximum feed rate. Next, a straight slot of length 23 mm was machined with the maximum feed rate in each experiment. Each experiment was performed twice, with the MRR being calculated each time. Finally, a sample was produced according to a preset CNC feed path using the experimental parameters giving the highest feed rate.

### Influence of diamond grain size

**Figure 7:**
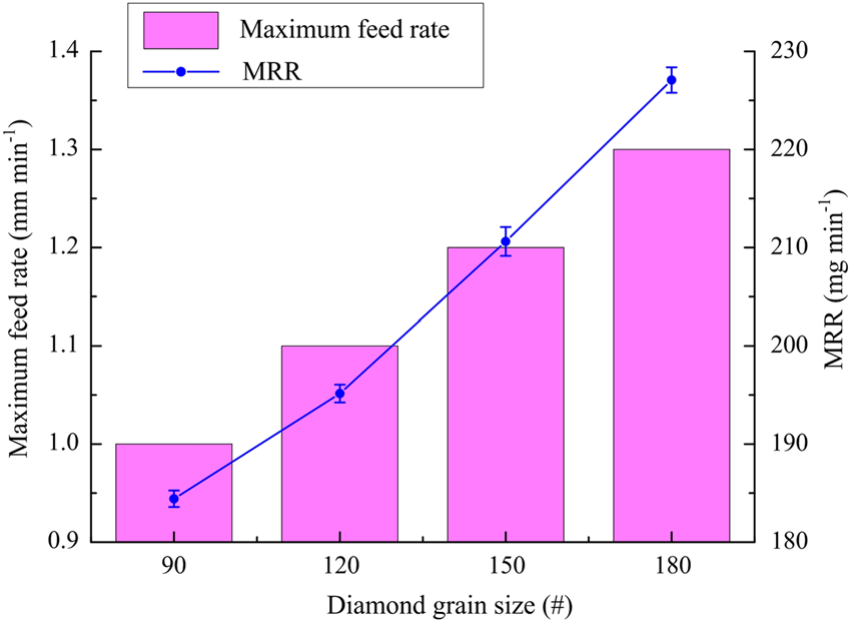
Variations of maximum feed rate and MRR with diamond grain size.

In this set of experiments, the applied voltage, electrolyte temperature and pressure were 15 V, 25 °C and 0.2 MPa, respectively. Figure 7 shows the variation of the maximum feed rate and MRR with diamond grit size. As the grain size increased from 90 to 18#, the maximum feed rate increased from 1.0 to 1.3 mm min^−1^.

**Figure 8:**
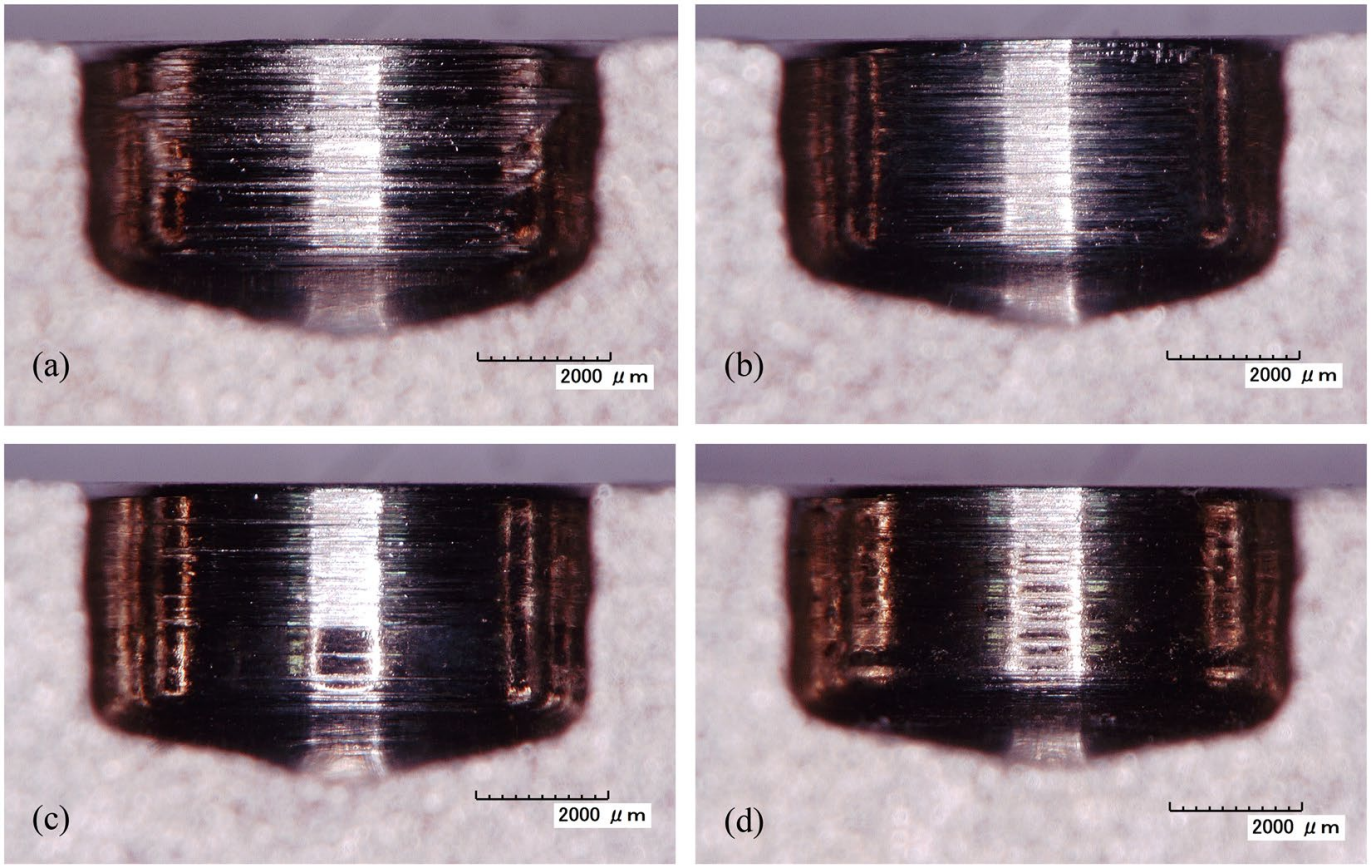
Cross-sections of machined slots for different diamond grain sizes: (**a**) 90#, 1.0 mm min^−1^; (**b**) 120#, 1.1 mm min^−1^; (**c**) 150#, 1.2 mm min^−1^; (**d**) 180#, 1.3 mm min^−1^.

In ECG, the machining gap is determined by the height by which the diamond particles protrude above the surface of the abrasive tool. Generally, as this height decreases, so does the machining gap, which may lead to greater electrochemical activity^22^. When a diamond abrasive tool is manufactured, the diamond grain size is inversely proportional to the mesh. Cross-sections of machined slots obtained for different grain sizes are shown in Fig. 8. It can be clearly seen that grinding marks become more noticeable as the diamond grain size increases. It can also be seen that the grinding action is weaker for smaller diamond grain size. Obviously, the maximum feed rate and the MRR are both increased when the electrochemical activity in ECG is enhanced. However, the grinding action may be restricted when the diamond grain size is too small. Therefore, diamond particles of the appropriate size should be used to achieve greater machining efficiency.

### Influence of applied voltage

**Figure 9:**
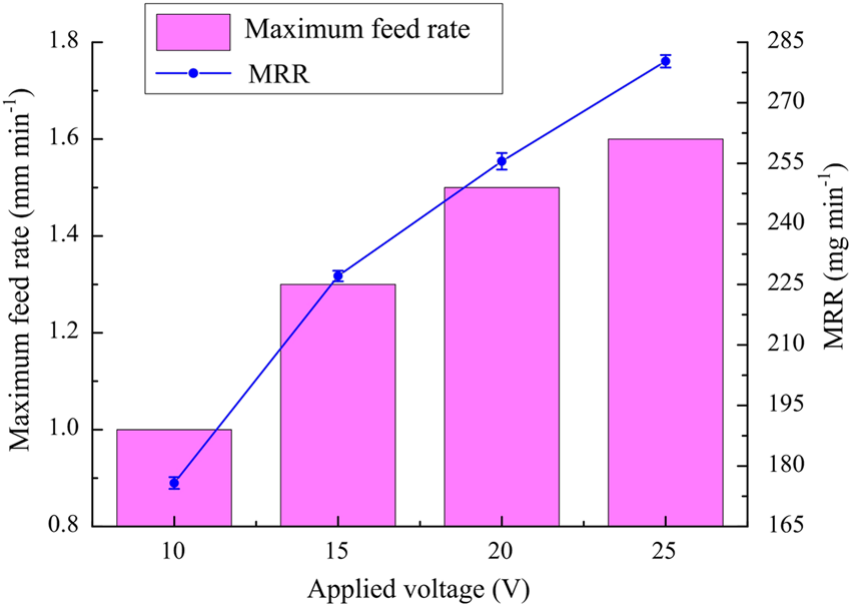
Variations of maximum feed rate and MRR with applied voltage.

In this pair of experiments, the diamond grain size, electrolyte temperature and pressure were 180#, 25 °C and 0.2 MPa, respectively. Figure 9 shows the variations of the maximum feed rate and MRR with applied voltage. As the applied voltage increased from 10 to 25 V, the maximum feed rate increased from 1.0 to 1.6 mm min^−1^.

**Figure 10:**
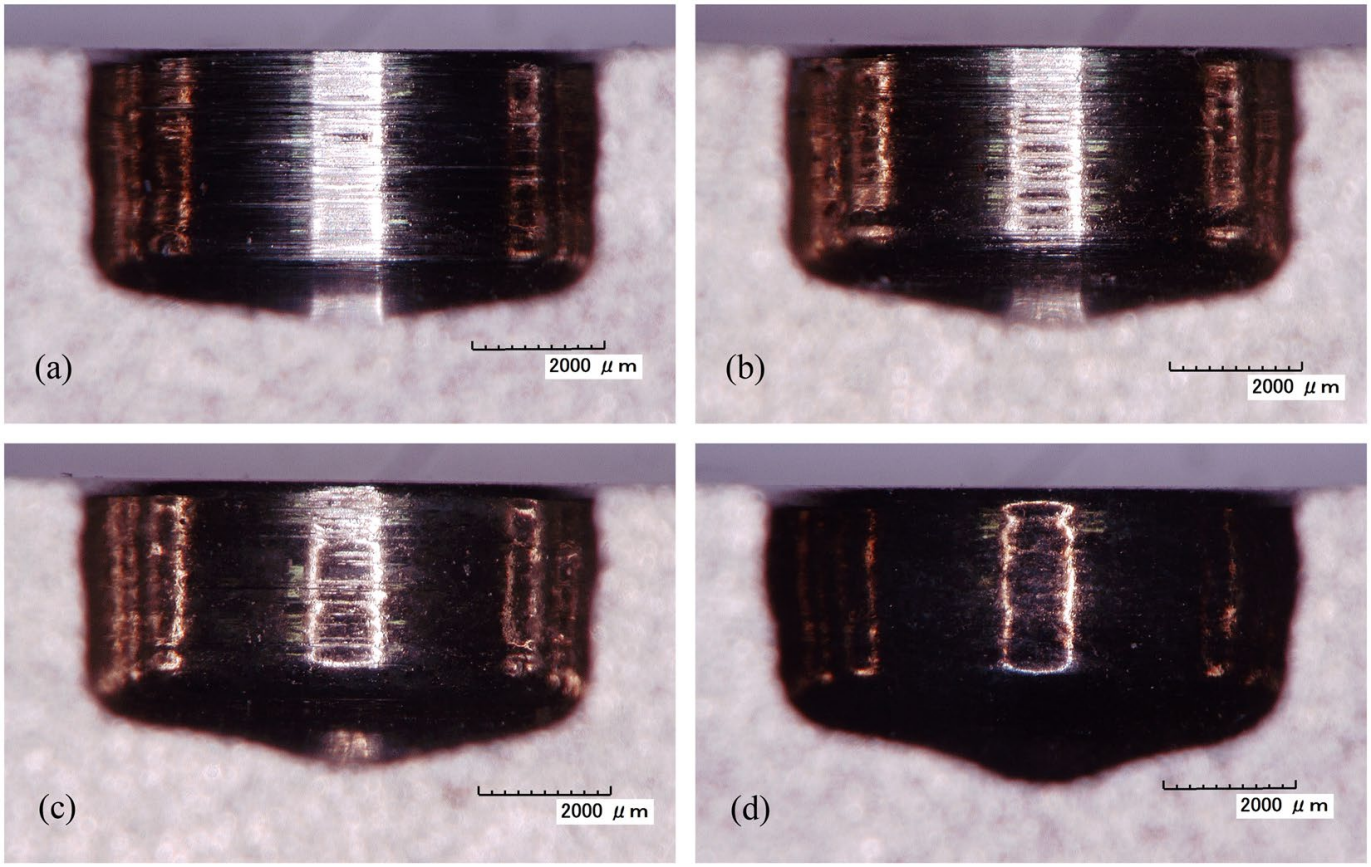
Cross-sections of machined slots at different applied voltages: (**a**) 10 V, 1.0 mm min^−1^; (**b**) 15 V, 1.3 mm min^−1^; (**c**) 20 V, 1.5 mm min^−1^; (**d**) 25 V, 1.6 mm min^−1^.

A passivation layer develops on the machined surface because a passive NaNO_3_ electrolyte is used^11, 13^. The use of a high applied voltage accelerates the formation of the passivation film, thereby greatly increasing the processing efficiency. Meanwhile, the machining gap becomes larger as the applied voltage is increased, owing to enhancement of the effects of electrolysis. Therefore, the feed rate should be further increased so as to improve MRR. Cross-sections of machined slots obtained at different applied voltages are shown in Fig. 10. Grinding marks are quite noticeable at an applied voltage of 10 V. The mechanical abrasive action plays a relatively significant role in this case because the applied voltage is too low. However, with increasing applied voltage, fewer and fewer grinding marks can be observed, owing to the gradual increase in electrochemical dissolution. However, the rate of growth of the maximum feed rate declines as the applied voltage is increased, because it becomes more and more difficult to completely wash away electrolytic products from the machining gap. This indicates that an appropriate high voltage should be applied in order to obtain a higher MRR.

### Influence of electrolyte temperature

**Figure 11:**
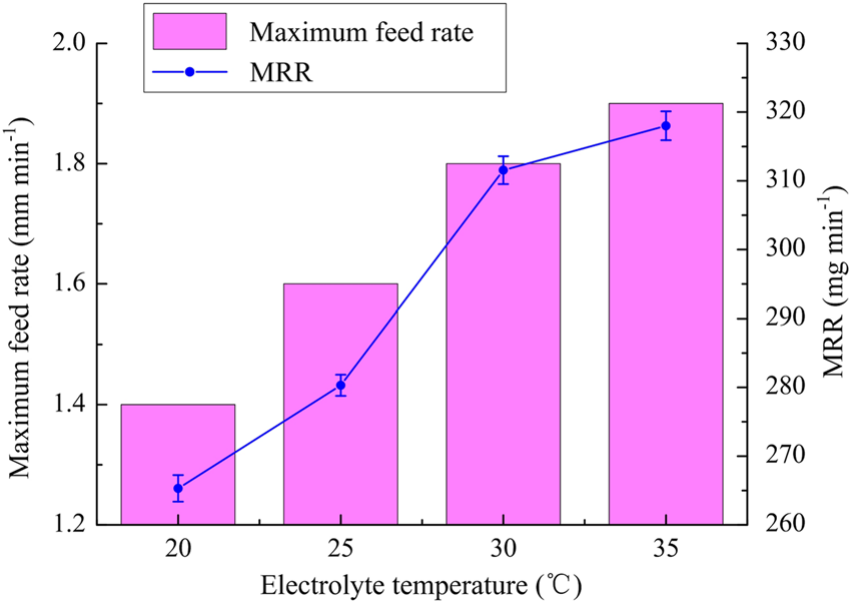
Variations of maximum feed rate and MRR with electrolyte temperature.

In this set of experiments, the diamond grain size, applied voltage, and electrolyte pressure were 180#, 25 V and 0.2 MPa, respectively. Figure 11 shows the variations of maximum feed rate and MRR with electrolyte temperature. As the electrolyte temperature increased from 20 to 35 °C, the maximum feed rate increased from 1.4 to 1.9 mm min^−1^.

Temperature is one of the key factors affecting the conductivity of the electrolyte. It is usually proportional to the electrical conductivity of the electrolyte and to the current density, because ions in the electrolyte become more active as the temperature increases. As a result, the maximum feed rate increases with increasing electrolyte temperature owing to enhancement of electrochemical dissolution, which also leads to increased MRR during the fabrication of a slot. However, when the electrolyte temperature becomes too high, the electrolyte in the neighbourhood of the machining gap can readily reach boiling point owing to Joule heating and to heat generated by grinding. This means that a sparker or short-circuit can easily arise owing to unsteady flow in the machining gap. Therefore, too high an electrolyte temperature should be avoided in inner-jet ECG. It can be concluded that the choice of correct electrolyte temperature can improve the efficiency of material removal.

### Influence of electrolyte pressure

**Figure 12:**
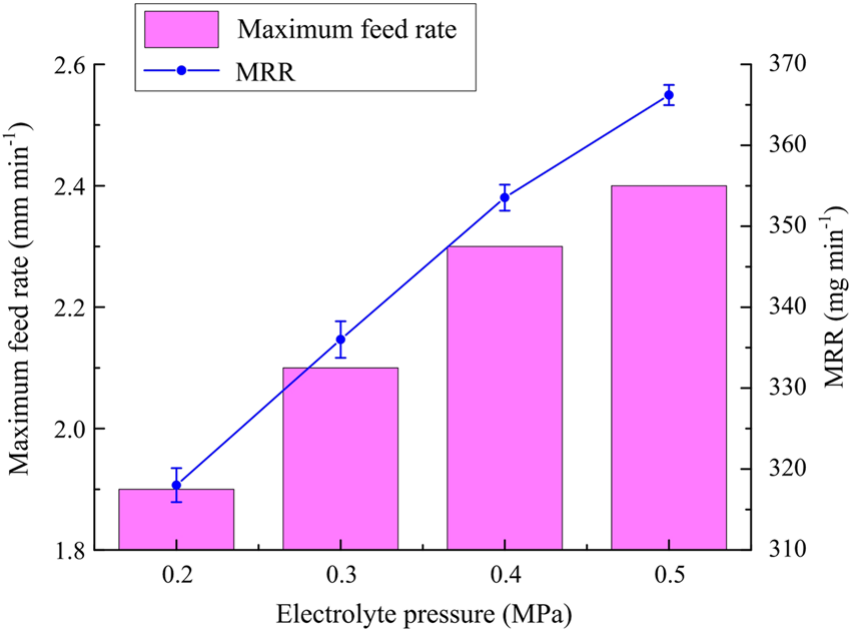
Variations of maximum feed rate and MRR with electrolyte pressure.

In this pair of experiments, the diamond grain size, applied voltage and electrolyte temperature were 180#, 25 V and 35 °C, respectively. Figure 12 shows the variations of maximum feed rate and MRR with electrolyte pressure. As the electrolyte pressure increased from 0.2 to 0.5 MPa, the maximum feed rate increased from 1.9 to 2.4 mm min^−1^.

With increasing electrolytic activity, a greater volume of products and a greater amount of heat are produced in the machining gap. Holes in the sidewall of the abrasive tool can easily become blocked by a large amount of insoluble electrolytic products if the electrolyte pressure is not high enough to wash them away. Meanwhile, the conductivity of the electrolyte gradually drops owing to more and more hydrogen being produced by electrolysis. In addition, electrolyte in the neighbourhood of the machining gap can reach boiling point because of the massive amount of heat generated by both electrolysis and grinding. These effects would have a deleterious effect on processing stability. Therefore, an increase in electrolyte pressure is useful because it enhances the removal of electrolysis products and heat from the machining gap, thereby enhancing the efficiency and stability of the ECG process.

With the machining depth set at 3 mm in one pass, a feed rate of 2.4 mm min^−1^ could be obtained using the proposed abrasive tool, which gave a higher removal efficiency than the round-ended tool used previously for inner-jet ECG^26^. In addition, although the feed speed of the workpiece can reach 200 mm min^−1^ during high-speed grinding of nickel-based alloy, the depth of cut was set to only 0.01 mm to avoid over-rapid wear of the abrasive grit^31^. From a comparison of the values of the product of machining depth and feed rate for inner-jet ECG and high-speed grinding, respectively, it is found that inner-jet ECG with the proposed abrasive tool has an obvious advantage in allowing a large depth of removal for difficult-to-cut materials.

### Sample fabrication

**Figure 13:**
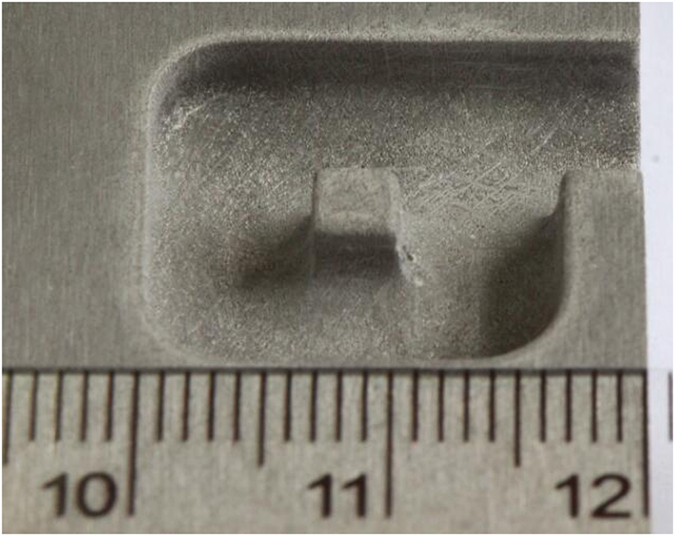
Photograph of machined sample.

The sample was produced along a predefined feed path with a feed rate of 2.4 mm min^−1^ and a machining depth of 3 mm. The total machining length was 47 mm and the feed direction was changed four times. The diamond grain size, applied voltage, electrolyte temperature and pressure were 180#, 25 V, 35 °C and 0.5 MPa, respectively. The processed sample is shown in Fig. 13. The surface roughnesses of the bottom and the sidewall of this sample, as obtained using a roughometer (Perthometer M1, Mahr, Germany), were 3.259 and 2.336 μm, respectively. It is clear that inner-jet ECG with the proposed abrasive tool has improved flexibility and extendibility for the machining of difficult-to-cut materials.

## Conclusions

The flow field during inner-jet ECG using an abrasive tool based on a dead-end tube has been analysed both for a flat-ended tube with electrolyte outlets located in the tool sidewall and for a round-ended tube with outlets in the end. The results of these flow-field simulations and of experimental investigations lead to the following conclusions:

1. The simulation results indicate that the uniformity of the flow field in the inter-electrode gap can be greatly improved by increasing the diameter and number of outlet holes in the sidewall of the flat-ended tube, although the associated decrease in the effective working area of the tool also has to be taken into account. The optimum choice was found to be six uniformly distributed outlet holes of diameter 1 mm. Compared with the round-ended tool tube, the flow velocity distribution is more uniform in the frontal gap.
2. The experimental results show that larger diamond grain size, applied voltage, electrolyte temperature and pressure are conducive to increasing the feed rate and MRR. With the flat-ended tube, a machining depth of 3 mm in a single pass at a feed rate of 2.4 mm min^−1^ was obtained with a diamond grain size of 180#, an applied voltage of 25 V, an electrolyte temperature of 35 °C and an electrolyte pressure of 0.5 MPa. The efficiency of material removal was improved significantly compared with that achieved with a round-ended tool tube.
3. Using the flat-ended tool tube, a sample was fabricated along a CNC feed path at a feed rate of 2.4 mm min^−1^ and a machining depth of 3 mm. The total length of machining was 47 mm and the direction of feed was changed four times. The surface roughnesses of the bottom and the sidewall of this sample were 3.259 and 2.336 μm, respectively. It was found that inner-jet ECG with the proposed abrasive tool is a promising method for machining GH4169 alloy into complex shapes with a large removal depth.

This investigation has demonstrated that inner-jet ECG with a dead-end tubular tool has high efficiency and good flexibility for processing hard-to-cut metals with a large removal depth. In future work, appropriate insulation will be incorporated at the bottom of the tool with the aim of improving the flatness of the machined bottom and the contribution of the grinding effect will be explored for different machining parameters.
